# Effect of drops containing *Lactobacillus reuteri* (DSM 17938 and ATCC PTA 5289) on plaque acidogenicity and other caries-related variables in orthodontic patients

**DOI:** 10.1186/s12866-021-02310-2

**Published:** 2021-10-06

**Authors:** Sahal Alforaidi, Andrea Bresin, Naif Almosa, Anna Lehrkinder, Peter Lingström

**Affiliations:** 1 Department of Cariology, Institute of Odontology, The Sahlgrenska Academy, University of Gothenburg, Gothenburg, Sweden; 2 Department of Pediatric Dentistry and Orthodontics, College of Dentistry, Taibah University, Medina, Saudi Arabia; 3 Specialist Clinic of Orthodontics, Public Dental Service, Region Västra Götaland, Gothenburg, Sweden; 4 Department of Orthodontics, Institute of Odontology, The Sahlgrenska Academy, University of Gothenburg, Gothenburg, Sweden; 5 Department of Pediatric Dentistry and Orthodontics, College of Dentistry, King Saud University, Riyadh, Saudi Arabia

**Keywords:** Dental biofilm, Dental plaque^.^ Lactobacillus reuteri (L. reuteri), Plaque pH, Probiotics^.^ Saliva, Streptococcus mutans (S.mutans), Quantitative polymerase chain reaction (qPCR)

## Abstract

**Background:**

The purpose of the study was to investigate the effect of probiotics on biofilm acidogenicity and on the number of salivary *Streptococcus mutans* and lactobacilli in orthodontic patients.

**Methods:**

This RCT was conducted on 28 young adults who were undergoing orthodontic treatment. The short-term prospective clinical trial lasted for three weeks. The test group rinsed daily with drops containing two *Lactobacillus reuteri* strains diluted in water, while the placebo group used drops without probiotics. The subjects were enrolled eight months since the beginning of orthodontic treatment. Plaque-pH, saliva and dental biofilm samples were obtained at baseline, one week and three weeks post intervention.

**Results:**

Twenty-seven subjects successfully completed the trial period, only one drop out in the test group. No side effects were reported. A statistically significant increase in plaque pH at three weeks post-intervention was found for the test group (*p* < 0.05), while insignificant changes in the pH value were found for the placebo group in comparison to baseline (*p* > 0.05). In addition, the AUC_7.0_ showed a significant difference at three weeks between the test and placebo (*p* = 0.00002). The three-week samples of stimulated whole saliva showed a statistically insignificant difference in the number of *S. mutans* and lactobacilli between the two groups (*p* > 0.05). The qPCR analysis showed the ability of the two strains to get colonized in the dental biofilm without a significant effect on the microbial counts.

**Conclusion/clinical implications:**

A mixture of *Lactobacillus reuteri* has the ability to reduce the pH fall at the three-week follow-up. However, the short-term use of probiotics does not appear to have an effect on the number of salivary *Streptococcus mutans* and lactobacilli in saliva and on the dental biofilm.

**Trial registration:**

Clinicaltrial.gov (Identifier: NCT04593017/ (19/10/2020)).

## Background

Dental caries is one of the chronic diseases most commonly affecting oral health. The disease occurs as a result of the interaction between cariogenic bacteria (such as *mutans streptococci* and *lactobacilli*), a diet rich in fermentable carbohydrates and a susceptible host, including the saliva secretion rate and buffering capacity over a period of time [1]. Subsequently, the acid production resulting from carbohydrate metabolism by cariogenic bacteria will reduce the environmental pH, which will lead to mineral loss [2]. In addition, a number of factors, such as socioeconomic status, biological factors and genetics, may also be regarded as important factors for dental caries [3, 4]. Different strategies, such as fluoride application, dietary modification and oral hygiene, have been suggested to diminish or reverse enamel demineralisation. Lately, the intake of live bacteria of human origin has been added as a preventive strategy. Moreover, antibiotics and antimicrobial treatment with chlorhexidine have attracted attention over the last few years. However, the use of wide-spectrum antibiotics and antimicrobial treatment may reduce the caries risk but will never eliminate it, which means that they must be taken at regular intervals over the long term [5]. The use of bacteriotherapy is a fairly new concept in preventive dentistry, despite its long-time use in the prevention and treatment of gastrointestinal diseases. *Lactobacillus reuteri* is one of the microorganisms that has been extensively reviewed and showed their ability to produce antimicrobial substances [6]. Although the full mechanisms of probiotic action are still poorly understood, it is believed that these microorganisms have the ability to compete with pathogenic bacteria known to be hazardous to the health, as well as local and systemic immunomodulation [7, 8]. Previous short-term studies have revealed a positive effect by different probiotic micro-organisms, resulting in a reduction in the number of mutans streptococci [9, 10]. Other clinical trials have tested the effect of probiotics with caries as the endpoint [11, 12]. Several vehicles, such as tablets, lozenges, chewing gum, cheese, yogurt and different bacterial strains, have been used. However, the best vehicle for probiotic delivery for different patient groups has yet to be identified. Orthodontic patients undergoing treatment with fixed appliances may require additional preventive strategies that might help to reduce the caries risk throughout the treatment progress and raise the standard care of the patients. Previous researchers have reported a significant increase in the number of cariogenic bacteria in the dental biofilm during orthodontic treatment following the placement of the appliances [13]. To date, studies investigating the probiotic effect on orthodontic patients at the level of the bacterial change are limited and not explored in any depth with conflicting results. In addition, drops as a vehicle for probiotic administration in patients wearing orthodontic appliances have never been used before. Our primary aim was therefore to investigate the short-term effect of probiotic drops on plaque acidogenicity among orthodontic patients. The secondary outcome was the effect of probiotics on the level of *Streptococcus mutans* and *lactobacilli* in the saliva. The null hypothesis is that the effect of probiotics would not differ from that in a placebo-treated control group.

## Materials and methods

### Participants

**Fig. 1:**
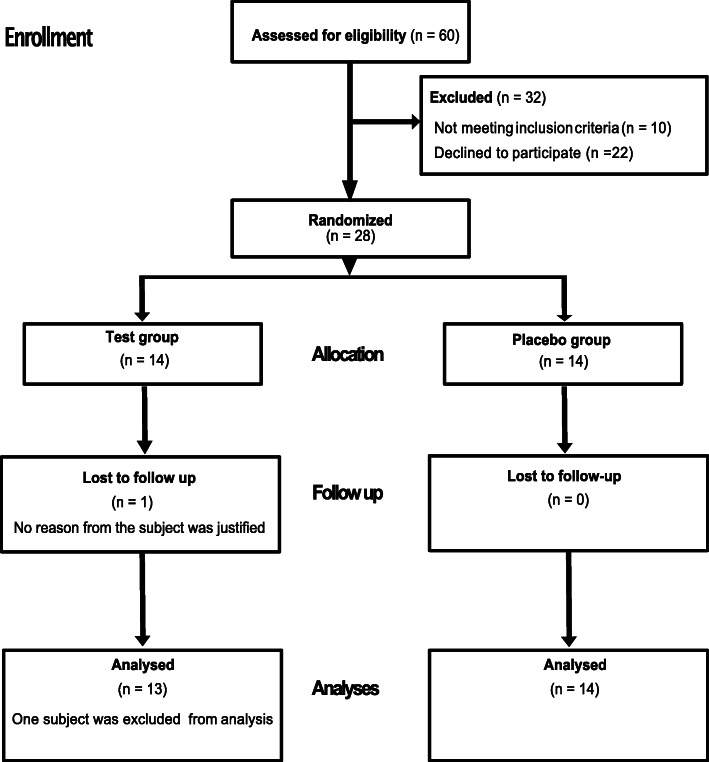
Flow chart of randomization and attrition of participants throughout the study period

The sample population consisted of 28 subjects, fourteen males and fourteen females, with a mean age of 17.3 ± 1.1 years, who were undergoing orthodontic treatment at the Specialist Clinic of Orthodontics, Public Dental Service, Gothenburg, Region Västra Götaland, Sweden. The sample size calculation was based on a significance level of 5 and 80% power in order to detect the difference in plaque acidogenicity with an estimated difference in pH fall of 0.4, SD 0.35. The power calculation revealed that, twelve subjects are needed in each arm, additional two subjects were added in each group accounting for the drop out. The inclusion criteria were subjects with a large number of *Streptococcus mutans* (> 10^4^ CFU/ ml of saliva), undergoing bimaxillary fixed orthodontic treatment, over a period of time lasting eight months since the onset of bonding, medically healthy, no medications, free of any systemic diseases and with the ability to understand the nature of the study. The exclusion criteria were patients with cleft lip and palate syndrome, handicapped patients, individuals with systemic diseases or conditions that could interfere with the study and a history of probiotics/anti-inflammatory drugs/antimicrobial substances taken during the last four weeks prior to the baseline examination. A saliva screening test was carried out to identify high caries risk subjects based on the number of *Streptococcus mutans* prior to study. Of 60 screened subjects, 28 were selected for the present study, ten subjects did not meet the inclusion criteria and twenty declined to participate. The subjects were stratified into the test and placebo groups based on gender and the level of *Streptococcus mutans* (Fig. 1). All the subjects had good oral health with no open or untreated carious lesions and a mean DMFT of 0.4 ± 0.8 and they claimed that they brushed their teeth twice a day.

The subjects were instructed to avoid other probiotic-containing products such as xylitol chewing gums and antibiotics during the study. The study protocol was in accordance with the Helsinki Declaration of Human Rights and was approved by the Ethics Committee at the University of Gothenburg (788–18). The subjects received both oral and written information about the study and gave their informed consent prior to the start of the study.

### Study design

The study has been registered at Clinicaltrial.gov with ID number NCT04593017/ (19/10/2020). The study was designed as a double-blind study, in which neither the investigator nor the participants were aware of the groups they were enrolled in. It was a placebo-controlled, randomised clinical trial with two parallel arms and comprised a three-week intervention following the CONSORT guidelines. The subjects were randomly assigned using an Excel program after stratification based on gender and the level of *S. mutans* performed by an independent member (A.L.) who worked as a microbiologist. Allocation was made to one of the two groups: A) test, mouth rinse using drops containing two strains of the probiotic bacterium *Lactobacillus reuteri*, and B) placebo, mouth rinse using identical drops without any active probiotic bacteria. The rinses were administered in the morning after breakfast and toothbrushing and in the evening before going to bed. Rinsing was at each time point carried out for 60 s after which the solution was spat out. No eating or drinking were allowed during the following two hours. The subjects were told to brush their teeth twice a day. For standardisation purposes, the same toothpaste (Folktandkräm, Proxident AB, Sweden) was distributed to all the participants.

### Study drops

The probiotic drops, BioGaia Prodentis drops (BioGaia AB, Stockholm, Sweden), contained freeze-dried *L. reuteri* DSM 17938 (> 1 × 10^8^ CFU/5 drops) and *L. reuteri* ATCC PTA 5289 (> 1 × 10^8^ CFU/5 drops) suspended in an oil [14]. The daily intake was 0.15 to 0.20 g (5 drops). The probiotic solution was prepared fresh by the test subject prior to each rinsing session by mixing five drops of a probiotic oil (*L. reuteri* DSM 17938 and *L. reuteri* ATCC PTA 5289) with 5 ml of distilled water according to the manufacturer instructions. Tubes were filled with 5 ml of distilled water and distributed to all subjects at each time point. During the study, the participants were instructed to keep the water and drops in a refrigerator when not in use. Compliance regarding the use of probiotics was checked using a special mobile app, MyMedschedule^R^ Plus, which reflects the mean percentage of mouth rinses with probiotics during the study period through a reminder which was given at a specific time every day.

### Clinical variables

The subjects came to the Department of Cariology, Institute of Odontology, for the collection of oral samples before the start of probiotic administration, one week post intervention and after three weeks’ administration of probiotic/placebo. The participants were asked to refrain from proximal cleaning for 48 h and toothbrushing for 24 h prior to each sampling session. On each visit, plaque pH, dental biofilm and saliva were assessed.

### Biofilm acidogenicity

Plaque pH was assessed using the strip method for all subjects and at all time points based on the technique described by Carlén et al [15], except for one subject in the test group in which pH was taken only at baseline as a result of drop out. A pH indicator strip (Spezialindikator, Merck, Darmstadt, Germany) was used to measure the pH value (4.0–7.0). Each strip was cut into three pieces and inserted in the interproximal area (under the contact point of the teeth) of the lateral incisors/canines in the left and right upper regions, before (0 min) and 2, 5, 10, 20 and 30 min after a one-minute mouth rinse with 10 mL of a 10% sucrose solution. In order to assess the pH values, comparisons were made between the colour appearing on the inserted strip and the index chart provided by the manufacturer. The two examiners (S.A. & A.A.) performing the pH measurements were assessed for inter-examiner reliability where the Kappa statistics revealed 0.93.

### Dental plaque and saliva sampling

Deep inter-proximal plaque samples were collected between the upper lateral incisors and canines on both sites using a sterile toothpick and then transferred immediately into microtubes with TA buffer for the detection and quantification of the probiotic strains under qPCR analysis.

Whole stimulated saliva was collected as previously described [16], where 5 ml of stimulated whole saliva was collected by getting the subjects to chew on 1 g of paraffin wax for five minutes and collected directly in a graded test tube, after which the secretion rate was calculated in ml/min. One ml was transferred to VMGII medium for microbiological analysis of the level of *S. mutans* and lactobacilli and 1 ml was also utilised for buffer capacity [17].

### Microbiological analysis of the saliva sample

All the samples were analysed at the Department of Cariology, the Institute of Odontology, Sahlgrenska Academy, University of Gothenburg, Sweden. After serial dilutions, counts of the salivary number of *Streptococcus mutans* and lactobacilli were evaluated using Mitis Salivarius agar (Sigma-Aldrich, NutriSelect®Plus, Germany) supplemented with 10% sucrose and bacitracin (0.2 U/ml) and Rogosa agar under anaerobic conditions at specific temperatures and incubation periods, as previously mentioned [14]. The number of colony-forming units were enumerated on the agar and for S. Mutans identified based on specific characteristic colony morphology. Rogosa agar is selective for all lactobacilli.

### Quantitative polymerase chain reaction (qPCR)

Identification and quantification were performed as previously described [14]. Briefly, prior to qPCR analysis, tubes with plaque samples were placed in a thermoshaker (TS-100C, Biosan SIA, Latvia) for 10 min at 95 °C and 1000 rpm to release genomic DNA. The qPCR relative quantification analysis was performed on an MIC analyser (Bio Molecular Systems, Upper Coomera, Australia). A conserved region (BAC16S) in the ribosomal gene, present in all bacteria, was used as the reference gene and both strain-specific and species-specific primers for *L. reuteri*, *S. mutans*, lactobacilli and streptococci were used as the gene of interest in the relative quantification analysis.

In total, the reaction mixture of 20 μl contained 1x qPCRBIO SyGreen mix (PCR BioSystems, London, UK), 400 nM of each forward and reverse primer (Sigma-Aldrich Co., LLC) and 2.5 μl (< 1 μg genomic) DNA template. All amplifications were carried out as duplicates in MIC tubes and caps (BioMolecular Systems, Upper Coomera, Australia). The relative expression software tool (REST) on the MIC analyser (Bio Molecular Systems, Australia) was used to perform the analysis. The details of each assay and primer sequence are presented in Table 1.

**Table 1:** Primer sequence and qPCR conditions used for relative quantification analysis

	Primer sequence 5′-3′	qPCR program
*Streptococcus mutans*	Forward: CTACACTTTCGGGTGGCTTG	95 °C 2 min,
Choi et al., 2009 [18]	Reverse: GAAGCTTTTCACCATTAGAAGCTG	40 × 95 °C 10s, 61 °C 20s, Plate read
Total bacteria (reference gene)	Forward: TGGAGCATGTGGTTTAATTCGA	94 °C 4 min,
Choi et al., 2009 [18]	Reverse: TGCGGGACTTAACCCAACA	40 × 94 °C 20s, 62 °C 20s, Plate read
Total lactobacillus	Forward: TGGAAACAGRTGCTAATACCG	98 °C 2 min,
Roy et al., 2004 [19]	Reverse: GTCCATTGTGGAAGATTCCC	40 × 98 °C 10s, 62 °C 15 s, Plate read
*Lactobacillus reuteri* DSM 17938	Forward: TTAAGGATGCAAACCCGAAC	98 °C 2 min,
Vestman et al., 2013 [20]	reverse: CCTTGTCACCTGGAACCACT	40 × 98 °C 5 s and 60 °C 15 s, Plate read
*Lactobacillus reuteri* PTA 5289	Forward: GACAGTGGCTAAACGCCTTC	98 °C 2 min,
Vestman et al., 2013 [20]	Reverse: AATTCCACTTGCCATCTTCG	40 × 98 °C 5 s and 60 °C 15 s, Plate read
Total Streptococci	Forward: YGTGCAATTTTTGGATAAT	95 °C 3 min,
Täpp et al., 2003 [21]	Reverse: TTCTATAAGCCATGTTTTGT	40 × 94 °C 20s, 52 °C 30s, Plate Read

### Statistical analysis

The mean pH of the pH readings for each individual site was calculated. The mean for the two sites at each of the different time points was calculated, as well as the minimum pH and maximum pH fall. The area under the curve (AUC_7.0_) was calculated using Prism software. The mean ± SD and 95% confidence interval for the salivary *S. mutans* and lactobacilli concentrations were calculated for each group.

The data analysis was processed and analysed with GraphPad Prism software (version 8.2.0 (272)). Two-way analysis of variance (ANOVA) followed by Tukey’s multiple comparisons test was used for the changes in the pH value within the test and placebo groups. Comparisons of changes in the plaque pH between the two groups were made using Sidak’s multiple comparisons test. Detailed information regarding the pH readings was reported using paired t-test. Salivary cariogenic bacteria between the test and control groups were assessed using a two-way ANOVA test. Changes in the buffer capacity between the two groups was tested using paired t-test. The relative quantification of investigated probiotic bacteria species in plaque was carried out using qPCR gene expression analysis (Bio Molecular Systems, Australia), a statistical analysis was carried out using one-way ANOVA followed by Tukey’s comparison test. A difference with a value of *p* < 0.05 was considered to be statistically significant.

## Results

Twenty-seven subjects successfully completed the trial period, only one drop-out subject in the test group was recorded after baseline, the compliance was high (99%) based on the use of the MyMedschedule^R^ plus app, the percentage expressed the mean of the overall use by all participants during the trial period. No negative side-effects were reported in either group.

At baseline, the plaque pH and the number of salivary *Streptococcus mutans* and lactobacilli did not show any statistically significant difference for the observed variables between the test and placebo groups (*p* = 0.1) (Tables 2 and 3).

**Table 2:** Comparison of the level of *S. mutans* and lactobacilli between the two groups at baseline, one week post intervention and three weeks post intervention. The data are expressed in terms of the mean, 95% confidence interval and level of significance at *p* < 0.05

	Mean difference	95% CI	*P* value
Baseline (t0)			
*S. mutans* test vs *S. mutans* placebo	0.066	−0.906 to 1.038	> 0.05
Lactobacilli test vs Lactobacilli placebo	−0.043	−1.064 to 0.978	> 0.05
One week (t1)			
*S. mutans* test vs *S. mutans* placebo	−0.098	−1.089 to 0.893	> 0.05
Lactobacilli test vs Lactobacilli placebo	0.221	−0.814 to 1.256	> 0.05
Three week (t2)			
*S. mutans* test vs *S. mutans* placebo	−0.081	−1.116 to 0.954	> 0.05
Lactobacilli test vs Lactobacilli placebo	0.204	−0,790 to 1199	> 0.05

**Table 3:** Information about pH measurements regarding baseline pH value, maximum pH fall, final pH, mean minimum pH value and the AUC at a critical value of pH 7. A comparison was made between the test and control group at the level of *p* < 0.05

	BASELINE			1 week			3 weeks		
	Test	Placebo	t-test	Test	Placebo	t-test	Test	Placebo	t-test
	Mean (±SD)	Mean (±SD)	p value	Mean (±SD)	Mean (±SD)	p value	Mean (±SD)	Mean (±SD)	p value
Baseline	6.85 (±0.37)	6.85 (±0.28)	> 0.999	6.93 (±0.15)	6.87 (±0.26)	0.474	6.95 (±0.13)	6.86 (±0.23)	0.227
Max pH drop	1.60 (±0.3)	1.35 (±0.32)	0.047	1.14 (±0.5)	1.37 (±0.29)	0.152	1.02 (±0.31)	1.26 (±0.42)	0.106
Final pH	6.14 (±0.44)	6.37 (±0.5)	0.217	6.56 (±0.43)	6.33 (±0.37)	0.147	6.67 (±0.34)	6.42 (±0.35)	0.072
Min- max	4.70–7.00	4.55–7.00		5.15–7.00	4.70–7.00		5.45–7.00	4.80–7.00	
Mean min pH	5.2 (±0.33)	5.4 (±0.39)	0.105	5.7 (±0.55)	5.5 (±0.34)	0.153	5.9 (±0.35)	5.5 (±0.41)	0.038
AUC	38.64 (±4.6)	31.96 (±4.79)	0.001	24.17 (±5.42)	31.31 (±4.25)	0.0007	19.27 (±4.44)	28.32 (±4.56)	0.00002

**Fig. 2:**
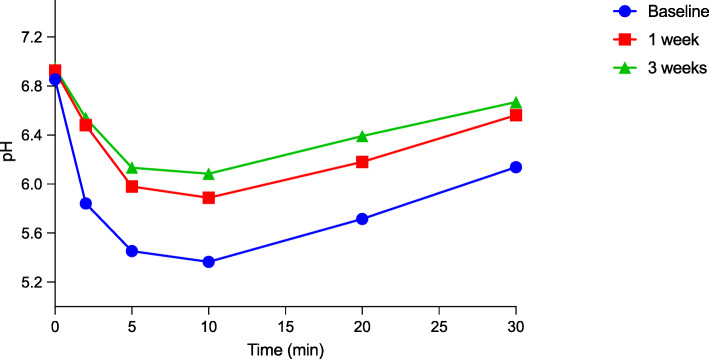
The curve shows the plaque-pH curves obtained at baseline, the one-week and the three-week follow-ups for the test group

**Fig. 3:**
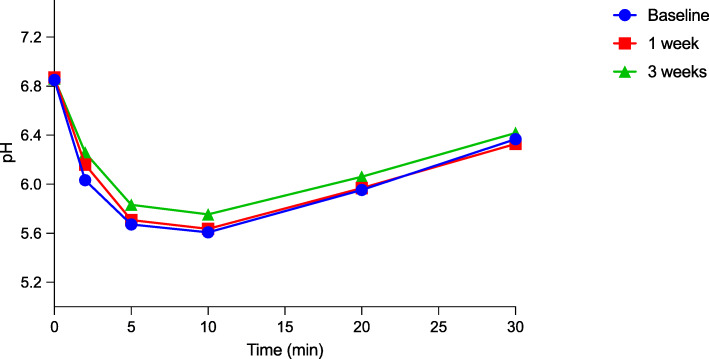
The curve shows the plaque-pH curves obtained at baseline, the one-week and the three-week follow-ups for the placebo group

Figures 2 and 3 show the plaque-pH curves obtained at baseline and the one-week and three-week follow-up for the test and placebo groups. For the test group, a statistically significant increase in plaque pH between baseline and the one-week follow-up and between baseline and the end of the intervention was found (*p* < 0.05). However, for the placebo group, there was no statistical difference at any of the time points.

Table 3 shows detailed information regarding the pH measurements. The results revealed a statistically insignificant difference between the test and placebo group based on the baseline pH value at all three time points (*p* > 0.05), but only the maximum pH fall was found to be significant at baseline (*p* < 0.05). Minor insignificant changes regarding the mean minimum pH value at baseline and at one week post intervention were seen, but, three weeks post intervention, the value started to be significant between two groups (*p* < 0.05). Furthermore, the values for the AUC_7.0_ between the test and placebo group were significant at baseline and one week post intervention. It is noteworthy that the largest difference seen when comparing the two groups was at the end of the intervention period (*p* = 0.00002).

**Fig. 4:**
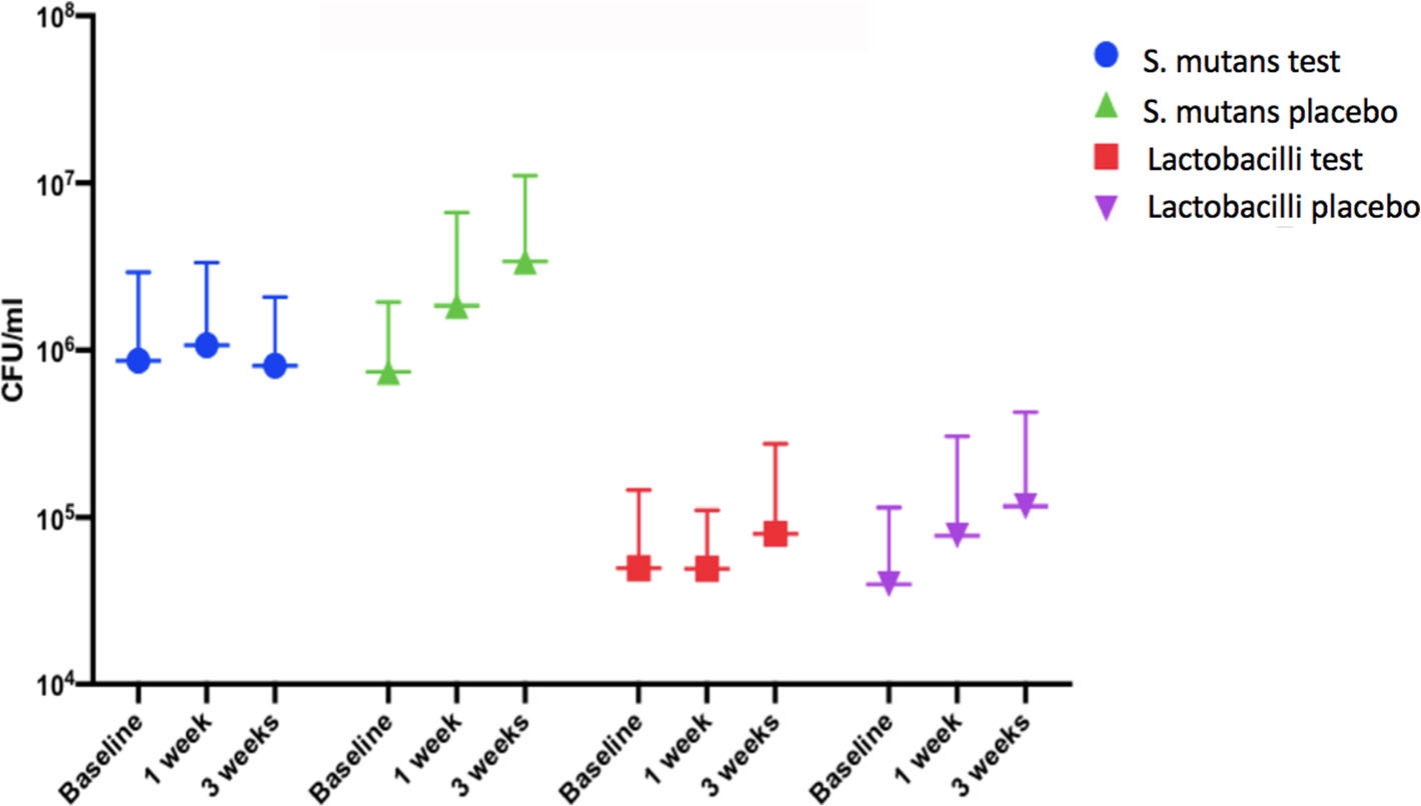
Salivary prevalence of S. mutans and total lactobacilli in the test and placebo groups at baseline, one week and three weeks post intervention. (*p* < 0.05)

Changes in the level of *S. mutans* and lactobacilli after probiotic use are presented in Fig. 4. No statistical difference was found for the salivary level of *S. mutans* and the level of lactobacilli between the test and placebo groups during the intervention period based on the cultural analysis (*p* > 0.05). It is worth noting that the level of salivary *S. mutans* and lactobacilli was more stable in the test group in comparison with the placebo group in which an increase was noted during the trial period. The overall salivary secretion rates for the individuals in the test and placebo groups were within normal with a mean for the three test visits of 1.78 ± 0.88 for the test group, and a mean of 1.50 ± 0.51 for the placebo group (ns). The results showed a numerical increase in buffer capacity for the test group (5.5 ± 1.1 for baseline, 5.9 ± 1.2 for 1 week and 6.0 ± 1.2 for 3 weeks; (ns), while no such trend was found for the placebo group (5.6 ± 1.2 for baseline, 5.7 ± 1.2 for 1 week and 5.6 ± 0.9 for 3 weeks).

The qPCR analysis of the dental biofilm showed that the two *Lactobacillus reuteri* strains, DSM 17938 and PTA 5289, were both detected for the test group at one week and at the three-week follow-up, with an increased value compared with baseline (*p* < 0.05), but both strains showed no effect on the total lactobacilli or total streptococci in relation to the total bacterial counts in the test and the placebo group (Table 4).

**Table 4:** Relative quantification of investigated bacteria species in plaque based on gene expression ratio. (*p* < 0.05)

***p***-value in relation to baseline		Week 1	Week 3
		Whole mouth	Whole mouth
***Lactobacillus reuteri*** **DSM/total lactobacilli**	**Test**	0.0015 (up)	0.0065 (up)
	**Placebo**	Too few samples	Too few samples
***Lactobacillus reuteri*** **PTA/total lactobacilli**	**Test**	0.028 (up)	0.009 (up)
	**Placebo**	Too few samples	Too few samples
**Total lactobacilli/total bacteria count**	**Test**	0.762 (no difference)	0.84 (no difference)
	**Placebo**	0.929 (no difference)	0.662 (no difference)
***Streptococcus mutans*/total streptococci**	**Test**	0.8305 (no difference)	0.986 (no difference)
	**Placebo**	0.644 (no difference)	0.549 (no difference)
**Total streptococci/total bacteria count**	**Test**	0.356 (no difference)	0.249 (no difference)
	**Placebo**	0.678 (no difference)	0.947 (no difference)

## Discussion

The effect of two strains of *Lactobacillus reuteri* administered twice daily on the plaque acidogenicity, on the level of salivary cariogenic bacteria and on the dental biofilm in a group of patients wearing orthodontic appliances in the short term was investigated. The novel aspect of this study is that, to date, the effect of probiotics on orthodontic patients has not been widely studied. In addition, pH and qPCR analyses are rarely used as methods when evaluating the outcome after probiotic administration.

It is worth noting that drops as a vehicle for probiotic administration have never been used on patients wearing orthodontic appliances. Hypothetically, this type of medium, when administered as a mouth rinse, may easily obtain access to the tooth surface around the wires and brackets. Other administrative modes, such as lozenges [22], yoghurt [23], probiotic curd and toothpaste [24], have previously been used by subjects undergoing orthodontic treatment.

The main outcome of this study was that probiotics in form of drops was significantly effective in reducing the plaque acidogenicity for the test group when compared to the placebo group after three weeks’ intervention. The null hypothesis could thus be rejected. Noteworthy, plaque acidogenicity was not considered as an outcome in any other clinical trials investigating the effect of probiotic on subjects wearing orthodontic appliances. Our study is in accordance with previous work in which probiotics was tested on high caries risk schoolchildren during a six week intervention period [25]. In contrast, the effect of probiotics lozenges on plaque pH has been investigated in young adults without orthodontic appliances. The study found no statistical significant difference between the test and placebo groups after two weeks of probiotics administration [26]. The short time period may explain these results, indicating the importance of continuous administration in order to attain the desired effect [27]. The results showed that, at baseline, both groups were matched. However, there was a significant difference in the maximum pH fall and in the area under the curve, which might be related to the inter-individual variations. For both parameters, the same pattern, in which the probiotic had more effect in the test group at the one-week and three-week follow-up, can be seen. The dental biofilm is regarded as a key factor in the caries process [28]. For this reason, a decrease in the plaque pH will result in the activation of the acidogenic bacteria, which may in turn result in significant tooth demineralisation [29]. Different tools, such as strips and electrodes, can be used for pH determination. In this case, the strip method was used as an equally effective tool reflecting plaque acidogenicity [15].

Most orthodontic patients suffer from retentive sites as a result of teeth irregularities and the presence of the orthodontic appliances which may contribute to the retention of *S. mutans* during the fixed orthodontic treatment [30]. This may in turn increase the risk of caries development and, in particular, the development of white spot lesions around the brackets [31]. In the present study, we found that drops containing two strains of probiotic *L. reuteri* did not help in reducing the number of *S. mutans* or the total number of lactobacilli in saliva after short-term use. However, contrary to the typical oral environment in orthodontic patients, the number of *S. mutans* and the total number of lactobacilli were fairly stable in the test group in comparison to the placebo group, in which a pronounced increase was noted. The present work is in agreement with previous work, in which the long-term use of probiotics in the form of lozenges administered once a day has no effect on the level of salivary *S. mutans* [22], whereas a significant reduction in the salivary mutans streptococci has been found in a group of orthodontic patients using probiotic yogurt containing *Bifidobacterium animalis* subsp. based on a chairside kit [10]. In a previous study using drops containing probiotics, our research team found a significant reduction in the number of *S. mutans* after four weeks of administration, where the oral environment is different from that in subjects wearing fixed appliances [14]. An interesting finding was that the buffer capacity increased during the three weeks for the test group in comparison to the placebo group. The exact explanation for the observed positive changes of biofilm acidogenicity cannot be explained by current data. Complex probiotic mechanisms suggested behind this action are normalisation of the oral microbiota, metabolic effects and modulation of the immune response [32], It is believed that the two first strategies play a central role in modulating pH.

The participants in the present study were enrolled eight months into their orthodontic treatment. The level of *S. mutans* and lactobacilli in the saliva and dental plaque has been shown to reach the highest level at this time point, which is regarded as an important factor for the risk of caries [33].

The results should be interpreted with caution for the following reasons: the short intervention period, the appropriate dose per day and the most optimal vehicle for probiotic administration.

Quantitative PCR using strain-specific and species-specific primer is a well-known method not only for detection but also as an accurate and reliable tool for bacterial quantification [34]. Plaque samples were collected between the upper lateral incisors and canines, as this area is the one most affected by caries in orthodontic patients [35–37]. In the present study, qPCR analysis indicated the ability of the probiotic to colonise the dental biofilm in the test group during the trial period, while no additional benefits at the level of the microbial effect could be seen. Few studies have investigated the effect of probiotics on microbial changes based on the PCR analysis. A study in which the effect of the probiotics in the form of systemic curd and toothpaste was tested on the dental biofilm found a significant reduction in *S. mutans* after 30 days in a group of orthodontic patients [24], while another study in the same group of patients found a similar effect when comparing the probiotic yogurt and the control yogurt on the reduction in mutans streptococci in both saliva and dental-plaque samples after short-term use [23]. There are different reasons for the conflicting results, such as the study design, the length of the trial period, the methods of microbial analysis, administration modes, age group, stage of orthodontic treatment and active probiotic strain. Additionally, although it is well known a large number of microorganism are involved in the caries process, only a limited number of highly relevant cariogenic microorganism were evaluated in the present study.

The results of our study call for long-term studies in this high-risk group to investigate whether probiotics can be regarded as an additional regimen in terms of caries prevention and to compare other strategies such as fluoride therapy and antibiotics in terms of side-effects and cost effectiveness.

## Conclusion

Probiotics appear to reduce the drop in plaque pH after a short period of use. However, it is unclear whether the effect of probiotics on pH will be maintained for a prolonged period of several months of orthodontic treatment with fixed appliances. If so, it may have a preventive effect on the development of carious lesions during orthodontic treatment. Further longitudinal studies of the prolonged use of probiotics in orthodontic patients and the development of carious lesions are needed to elucidate those hypotheses.

## Data Availability

All the primer sequences were referenced to a published works by Vestman et al., https://pubmed.ncbi.nlm.nih.gov/23486236/, https://pubmed.ncbi.nlm.nih.gov/25946126/ . Probiotics bacteria L. reuteri was obtained from the manufacturer (Biogaia, Stockholm) as commercial strains. There is no data to deposit in a database. No sequencing has been performed.

## Abbreviations

ANOVA: Analysis of Variance
AUC_7.0_: Area under the curve below pH 7.0
CFU: Colony-forming units
DNA: Deoxyribonucleic Acid
DMFT: The sum of the number of Decayed, Missing and Filled Teeth in the permanent dentition
L. reuteri: Lactobacillus reuteri
Max: Maximum
Min: Minimum
ns: Non-significant
pH: Potential of hydrogen or power of hydrogen
qPCR: Quantitative polymerase chain reaction
RCT: Randomized Clinical trial
S. mutans: Streptococcus mutans
SD: Standard deviation
TA buffer: Tris Acetate Buffer
VMGII medium: Viable medium by Gothenburg
